# Review of Journal of Cardiovascular Magnetic Resonance 2015

**DOI:** 10.1186/s12968-016-0305-7

**Published:** 2016-11-15

**Authors:** D. J. Pennell, A. J. Baksi, S. K. Prasad, R. H. Mohiaddin, F. Alpendurada, S. V. Babu-Narayan, J. E. Schneider, D. N. Firmin

**Affiliations:** Cardiovascular Magnetic Resonance Unit, Royal Brompton & Harefield NHS Foundation Trust, Sydney Street, London, SW 3 6NP UK

## Abstract

There were 116 articles published in the Journal of Cardiovascular Magnetic Resonance (JCMR) in 2015, which is a 14 % increase on the 102 articles published in 2014. The quality of the submissions continues to increase. The 2015 JCMR Impact Factor (which is published in June 2016) rose to 5.75 from 4.72 for 2014 (as published in June 2015), which is the highest impact factor ever recorded for JCMR. The 2015 impact factor means that the JCMR papers that were published in 2013 and 2014 were cited on average 5.75 times in 2015. The impact factor undergoes natural variation according to citation rates of papers in the 2 years following publication, and is significantly influenced by highly cited papers such as official reports. However, the progress of the journal's impact over the last 5 years has been impressive. Our acceptance rate is <25 % and has been falling because the number of articles being submitted has been increasing. In accordance with Open-Access publishing, the JCMR articles go on-line as they are accepted with no collating of the articles into sections or special thematic issues. For this reason, the Editors have felt that it is useful once per calendar year to summarize the papers for the readership into broad areas of interest or theme, so that areas of interest can be reviewed in a single article in relation to each other and other recent JCMR articles. The papers are presented in broad themes and set in context with related literature and previously published JCMR papers to guide continuity of thought in the journal. We hope that you find the open-access system increases wider reading and citation of your papers, and that you will continue to send your quality papers to JCMR for publication.

## Technical developments

Technological progress marches on and the cardiovascular system inspires considerable activity, because it is challenging and new applications become possible as acquisition speed increases. Recent advances reported in the journal have included improved flow mapping, [1–3] real-time imaging, [4] T1 mapping, [5–7] simulations, [8, 9] perfusion, [10–12] coronary imaging, [13] diffusion tensor imaging, [14–16] feature tracking, [17–20] and strain assessment. [21, 22] The full breadth of new developments is described in the papers in the section.

### Validation of in vivo 2D displacements from spiral cine DENSE at 3T

This manuscript describes a study to investigate the potential improvement of spiral cine DENSE when applied at 3 Tesla [23]. The study showed that the higher field strength and associated longer T1 resulted in higher SNR and tag persistence which allowed scanning at a higher resolution. The authors show increases in radial strain with this increased spatial resolution and in keeping with comparative lower field measurements, that reproducibility for radial strain is less than for the other measures.

### Quantitative pixel-wise measurement of myocardial blood flow. The impact of surface coil-related field inhomogeneity and a comparison of methods for its correction

As myocardial perfusion quantitation by CMR is being used more frequently this work represents important evidence that will improve the accuracy of techniques applied [24]. The results are highly relevant to the quantitative analysis of myocardial perfusion CMR studies. The authors show in volunteers that correction of perfusion CMR data with surface coil intensity correction improves homogeneity of myocardial blood flow estimates across the myocardium. They then show in patients that uncorrected estimates can lead to misclassification of perfusion defects. They conclude with the important message that surface coil-related field inhomogeneity can confound pixel-wise myocardial perfusion quantification.

### FLASH proton density imaging for improved surface coil intensity correction in quantitative and semi-quantitative SSFP perfusion cardiovascular magnetic resonance

On a related theme this manuscript demonstrated an improved approach to acquiring the coil correction data for quantitative perfusion images acquired using the SSFP sequence [25]. The work was based on the hypothesis that the off resonance response of the conventionally used low flip angle SSFP was very different to the higher flip angle sequence used to acquire the perfusion data and that this introduced errors. To overcome this problem the authors implemented a FLASH proton density sequence for the coil correction images, which was insensitive to off resonance affects. The authors demonstrated the method on 10 normal subjects and also in 10 patients where they showed improved coefficients of variation over the whole ventricle for quantitative myocardial perfusion and semi quantitative measures of perfusion.

### Age and gender-related normal left ventricular deformation assessed by cardiovascular magnetic resonance feature tracking

The method of Feature Tracking (FT) is becoming more and more widely applied as an approach to measurement of myocardial strain. This is in part because it can easily be retrospectively applied to routinely acquired cine images and in part because the other methods of strain measurement have proved difficult to implement. This manuscript has provided the first comprehensive set of reference values for CMR feature tracking imaging across a large age range [26]. It remains unclear exactly how FT measures relate to true myocardial strain.

### Comparison of diffusion tensor imaging by cardiovascular magnetic resonance and gadolinium enhanced 3D image intensity approaches to investigation of structural anisotropy in explanted rat hearts

This paper presents an interesting large piece of work comparing two methods of obtaining microstructural information from ex vivo myocardial tissue: diffusion tensor imaging; and a novel technique which derives a structural tensor [27]. The structural tensor is obtained from high-resolution T1-weighted imaging of fixed tissue perfused with gadolinium contrast agent during fixation. The contrast agent lies within myocardial shear layers, which are visible in the high-resolution CMR data. The authors conclude that the structural tensor framework is reliable, robust and the preferred option for myolaminar measurement in fixed myocardium. The approach may well be of use in future assessment of DTI developments.

### User-initialized active contour segmentation and golden-angle real-time cardiovascular magnetic resonance enable accurate assessment of LV function in patients with sinus rhythm and arrhythmias

This work combines a previously described real-time cardiac imaging sequence with an easy-to-use analysis tool based on user-initialized active contours for segmentation of the LV endocardium to extract the LV volume for each image frame [28]. The goal of the paper was to provide evidence that real-time CMR can accurately assess beat-to-beat variation in LV function or during an arrhythmia. In sinus rhythm patients, continuous LV volume measurements showed no significant difference compared to clinical standard segmentation of retrospectively-gated cine images and in arrhythmia, the impact of ectopic beats on hemodynamic function was demonstrated.

### Impact of motion correction on reproducibility and spatial variability of quantitative myocardial T2 mapping

In this study, the authors evaluated the impact of their own previously published in-plane motion correction algorithm; adaptive registration of varying contrast-weighted images for improved tissue characterization (ARCTIC), in quantitative myocardial T2 mapping [29]. They tested their approach on twelve healthy adult subjects imaged using breath-hold, free breathing, and free breathing with respiratory navigator gating for myocardial T2 mapping sequences. Additionally fifty patients referred for clinical CMR were imaged using the free breathing with respiratory navigator sequence. Their approach led to increased DICE scores, improved reproducibility and improved subjective score of T2 map quality. The authors conclude by saying their technique “substantially reduces spatial mis-alignment among T2-weighted images and improves the reproducibility and spatial variability of in-vivo T2 mapping”.

### Clinical experience of strain imaging using DENSE for detecting infarcted cardiac segments

In this study into the potential clinical application of DENSE, the authors tested its potential to uncover early systolic changes not picked up by ejection fraction [30]. They aimed to assess whether strain obtained by strain-encoded CMR with DENSE, could discriminate patients with myocardial scar. The main result were that circumferential strain allows the detection of segments with more than 50 % of myocardial scar and that interobserver and scan-rescan reproducibility was high. The conclusions were that DENSE-derived circumferential strain may be used for the detection of myocardial segments with >50 % scar area, the repeatability of strain is satisfactory and that DENSE derived global strain agrees with other global measures of left ventricular ejection fraction.

### Cardiovascular magnetic resonance compatible physical model of the left ventricle for multi-modality characterization of wall motion and hemodynamics

Dynamic wall motion as well as the hemodynamics of mitral inflow and aortic outflow are all simulated [31]. Chamber morphology is studied using stereo-photography and CMR and intra-chamber flow is evaluated using particle image velocimetry flow probes and phase contrast CMR. CMR and stereo-photography closely agree for volume assessment and centre plane flows from the two measures matched. The authors claim that the model can be used for the purposes of acquiring CMR data for validation of fluid-structure interaction simulations, determining the accuracy of cine-CMR reconstruction methods, and conducting investigations of the effects of altering anatomical variables on LV function under normal and disease conditions.

### Magnetic susceptibility anisotropy of myocardium imaged by cardiovascular magnetic resonance reflects the anisotropy of myocardial filament alpha-helix polypeptide bonds

This paper lies at the intersection of biophysics and magnetic resonance imaging [32]. The authors describe an interesting investigation of the anisotropic susceptibility of the tissue of the ex vivo murine heart. This yields the important finding that the magnetic susceptibility of heart muscle is anisotropic, such that the orientation of the principal axis of the susceptibility tensor is linked to the muscle fiber orientation. The measured values of susceptibility can be reasonably explained by using a simple compartmental model in which the anisotropy arises from the oriented peptide bonds in the myofibrils. The results are interesting from a basic science point of view and despite the many challenges also potentially point to diagnostic utility of cardiac susceptibility measurements.

### Influence of phase correction of late gadolinium enhancement images on scar signal quantification in patients with ischemic and non-ischemic cardiomyopathy

In this study the authors sought to investigate the existence of a systematic bias between threshold-based scar quantification performed on conventional magnitude inversion recovery (MIR) and matched phase sensitive inversion recovery (PSIR) images [33]. They carried out their study on 80 consecutive patients, 40 with ischemic and 40 with non-ischemic myocardial fibrosis and also 40 normal volunteers. They found that MIR images, compared with identical analysis of matched PSIR images, have relatively higher quantified scar signal using SD from the mean of normal, reference myocardium for Signal Threshold Versus Reference Myocardium techniques, and relatively lower quantified scar signal using full width half maximum analysis. The authors conclude that the systematic bias between MIR and PSIR based scar quantification suggests these two techniques should not be considered equivalent for the purposes of quantitative evaluation.

### Fast assessment of long axis strain with standard cardiovascular magnetic resonance: a validation study of a novel parameter with reference values

In this manuscript, the authors present a fast technique for the quantification of global LV longitudinal strain from standard CMR cine images [34]. Different approaches for the measurement of LV longitudinal strain were tested retrospectively in 125 patients with various forms of cardiomyopathy as well as in 40 controls. Strain derived from the distance of the epicardial apical border to the midpoint of the line connecting the mitral valve insertion points proved to be the most reliable approach. The method performed similarly to Feature Tracking for discriminating controls from patients and was considerably quicker. Additionally, the approach performed significantly better than MAPSE and the measurement of ejection fraction.

### Cardiovascular magnetic resonance phase contrast imaging

With the recent increased interest in CMR 4D Flow developments there has been relatively little attention more general 2D and developments for more routine clinical flow measurement applications. This review of phase-contrast CMR nicely addresses this limitation [35]. The manuscript includes an educational technical description of the technique mainly aimed at the more technically minded with a background in other CMR techniques and also includes a review of clinical applications and emerging research.

### Systolic ShMOLLI myocardial T1-mapping for improved robustness to partial-volume effects and applications in tachyarrhythmias

A real problem for the measurement of T1 by CMR is encountered when patients or subjects have arrhythmia. This paper looks to address the issue of assessment of T1 mapping in tachyarrhythmia by investigating whether images can be acquired in systole for this [36]. T1 maps were acquired using ShMOLLI at 1.5T in 10 healthy volunteers with various systolic trigger delays and also a conventional diastolic delay. A shorter readout was also implemented, to explore the effect of reducing image readout time and sensitivity to systolic motion. The feasibility and image quality of systolic T1 mapping was tested in 15 patients with tachyarrhythmia. The results showed reduced T1 variability in normals and in patients with tachyarrhythmia, systolic ShMOLLI T1-mapping was shown to overcome mis-triggering problems. For quantification there was a small overall effect on T1 values, with slightly shorter T1 values in systole compared to diastole.

### The effect of high-permittivity pads on specific absorption rate in radiofrequency-shimmed dual-transmit cardiovascular magnetic resonance at 3T

A particular problem that can affect CMR at 3T is the issue of B1 field inhomogeneity. This work analyses the effects on B1 homogeneity and SAR when a pair of high permittivity (HP) pads are used in combination with a commercial dual-cannel TX setup [37]. In a previous work the same group presented the design and optimization of the HP pads. The current work extends the concept through a further evaluation of B1 homogeneity and SAR for different B1 shim settings (amplitude and phase differences between channels) without and with the HP pads. They show that the combination of active (dual transmit) and passive (HP pads) RF shimming generally increases image quality for cardiac imaging at 3 T. In most practical cases optimized RF shim settings result in increased B1 and homogeneity and reduced SAR with the HP pads. However, there are cases in which SAR might be underestimated which is concerning for safety and indicates the need further investigation.

### Saturation pulse design for quantitative myocardial T1 mapping

This excellent paper highlights the importance of assessing, controlling and improving underlying basic elements of CMR acquisition sequence parameters that have a significant impact on the validity of findings in clinical studies [38]. The authors present a thorough analysis of improved saturation pulse designs for SASHA T1 mapping. They include 1.5T and 3T field strengths, and include the two important classes of pulses. They also characterize the connection between saturation imperfections and T1 errors. The authors conclude that Adiabatic and Pulse Train Saturation pulses optimized for different constraints found at 1.5T and 3T achieved <1 % residual |MZ/M0| in phantom experiments, enabling greater accuracy in quantitative saturation recovery T1 imaging.

### Validation of high temporal resolution spiral phase velocity mapping of temporal patterns of left and right coronary artery blood flow against Doppler guidewire

In this manuscript, the authors describe a prospective study comparing velocities measured using a novel spiral phase contrast velocity CMR sequence to those measured by Doppler guidewire, in 8 right and 15 left coronary arteries [39]. The paper describes the validation of a novel spiral PCMR sequence for use in coronary arteries and proposes the technique as a non-invasive alternative to Doppler. The results show that when corrected for differences in heart rate between the two studies, MR mean velocity through the cardiac cycle, peak systolic velocity (PSV) and peak diastolic velocity (PDV) showed a moderate to good linear relationship between the two techniques. MR values of PDV/PSV showed a strong linear relationship with Doppler values with a slope close to unity. The authors conclude that high temporal resolution breath-hold spiral phase velocity mapping underestimates absolute values of coronary flow velocity but allows accurate assessment of the temporal patterns of blood flow.

### Gadolinium free cardiovascular magnetic resonance with 2-point Cine balanced steady state free precession

Recently, there has been particular interest in the development of non-contrast alternatives to the routinely applied contrast enhanced CMR sequences [40]. In this work, the idea that Magnetization Transfer (MT) sensitivity of the balanced SSFP signal can be used to characterize the myocardium is revisited in a refined form. The authors present their technique based on repeating the sequence with different flip angles for imaging of scar and edema. The technique was evaluated in patients and showed good correlation with LGE. In the 23 who demonstrated areas of myocardial enhancement with LGE the association between LGE and their measure ΔS/So was strong. Bland-Altman analysis revealed a slight bias towards larger volume of enhancement with ΔS/So compared to LGE, and similar transmurality. The authors concluded that the method identified tissue that enhances at LGE with strong association to GPC and their results suggest that with further development, MT-weighted CMR could be used similarly to LGE for diagnostic imaging.

### 2D cine DENSE with low encoding frequencies accurately quantifies cardiac mechanics with improved image characteristics

This note reports the effect of the displacement encoding strength on the measurement of displacement and strain using DENSE phase encoded myocardial function evaluation [41]. Spiral DENSE is becoming established as a good method of characterizing regional myocardial strain. In this work acquisitions at 3T in healthy volunteers and patients, at various encoding strengths were performed and compared. Circumferential and radial strain and ventricular twist were evaluated. The conventionally used encoding strength of 0.1 cycles/mm has been shown result in significant phase wrapping that significantly complicates the processing. This study showed that for 2D cine DENSE with through-plane dephasing, the encoding frequency can be lowered to 0.04 cycles/mm without compromising the quantification of twist or strain.

### Breath-hold imaging of the coronary arteries using quiescent-interval slice-selective (QISS) magnetic resonance angiography: pilot study at 1.5 Tesla and 3 Tesla

In this study, the authors describe the adaption of the Quiescent-Interval Slice-Selective (QISS) noncontrast MRA method from peripheral MRA to breath-held coronary MRA [42]. The technique was tested at 1.5T and 3T and with both radial and Cartesian k-space trajectories. The left coronary system was imaged in 6 normal volunteers and 1 patient with mild coronary artery disease, and compared to a breath-held T2-prep 2D sequence and a standard 3D free-breathing bSSFP sequence. The QISS sequence is a single-shot or two-shot 2D sequence with a slice-selective inversion recovery preparation that suppresses background tissue. This method can be used to collect 10-20 slices in a long breath-hold, although less slices with a shorter breath-hold may be more suitable for patients. Results showed that overall, the image quality with the breath-hold multislice 2D QISS technique is very impressive and compares visually favorably with the free-breathing 3D coronary bSSFP scan. Cartesian QISS provided the best coronary-to myocardium CNR, whereas radial QISS provided the sharpest coronary images. QISS image quality exceeded that of free-breathing 3D coronary MRA with few artifacts at either field strength. The authors conclude that, with further clinical validation, QISS might provide an efficient alternative to commonly used free-breathing coronary MRA techniques.

### Parallel simulations for QUAntifying RElaxation magnetic resonance constants (SQUAREMR): an example towards accurate MOLLI T1 measurements

In another study towards improving the accuracy of T1 mapping, the authors have incorporated a relaxation time sequence simulator based on a general sequence simulator that had been described previously [43]. Using this the authors studied the improvement in accuracy for T1 mapping when using a strategy for determining T1 based on imaging signal intensities and simulations of the relationships between signals and sequence acquisition parameters. The proposed strategy was evaluated in phantoms compared to reference techniques and also in four healthy volunteers. T1 mapping schemes were performed where for every experiment, the identical pulse sequence was simulated for a large range of physiological combinations of relaxation constants, resulting in a database of all possible outcomes. The unknown relaxation constants were then determined by finding the simulated signals in the database that produced the closest fit to the measured signal intensities. The authors believe the method will allows for correction of quantitative CMR data that have already been acquired as well as improving data consistency and advancing quantitative CMR across imaging centers.

### Segmented nitinol guidewires with stiffness-matched connectors for cardiovascular magnetic resonance catheterization: preserved mechanical performance and freedom from heating

In the field of Interventional CMR there has been a real need for a guidewire that is mechanically strong, is trackable, and has the required bend along its length. It also needs to be safe in terms of potential for RF heating. The passive guidewire described in this study is a segmented-core nitinol device constructed using short nitinol rod segments [44]. Mechanical integrity was tested according to International Organization for Standardization (ISO) standards. CMR RF heating safety was tested in vitro in a phantom according to American Society for Testing and Materials (ASTM) F-2182 standard, and in vivo in seven swine. Results were compared with a high-performance commercial nitinol guidewire. The guidewire exhibited similar mechanical behavior to the commercial comparator. RF heating was reduced from 13 °C in the commercial guidewire to 1.2 °C in the described wire, using a flip angle of 75°. The guidewire was conspicuous during left heart catheterization in swine. The authors finally conclude that clinical translation is imminent.

### Positive contrast spiral imaging for visualization of commercial nitinol guidewires with reduced heating

Based on the lack of novel MR compatible guidewires, in this work the same group have also studied the development of sequences for safe use with conventional commercial guidewires [45]. For improved catheter visualization positive contrast was achieved using through-slice dephasing such that the guidewire appeared bright and the background signal suppressed. Positive contrast images were interleaved with anatomical images, and real-time image processing was used to produce a color overlay of the guidewire on the anatomy. Left heart catheterization was performed in a porcine model with a frame rate of over 6 frames per second. In comparison to the conventional Cartesian real-time imaging sequence, temperature increases reduced from 50 °C (phantom) and 4 °C (in vivo) to 0.37 °C (phantom) and 0.06 °C (in vivo). Visualization and localization of interventional devices (especially guidewires) using CMR is one of the challenging issues that are currently holding interventional CMR from becoming a clinical reality.

## T1 Mapping and ECV

There has been significant progress in developing standardised protocols of acquisition and analysis for T1 mapping. Its research use is still growing [46]. Much remains to be achieved in converting these efforts into substantive clinical applications, although the first of these looks to be in the assessment of cardiac amyloidosis.

### Single bolus versus split dose gadolinium administration in extra-cellular volume calculation at 3 Tesla

There is established evidence that ECV calculated using either an infusion or bolus of contrast agent is reproducible, and correlates well with fibrosis measured on myocardial biopsy specimens. However an area of contention is whether a split dose of contrast as is commonly used in adenosine stress perfusion protocols affects ECV and how this correlates with previously validated methods. In this study of 5 patients and 10 healthy volunteers, split dose contrast T1 mapping, in keeping with a stress perfusion protocol, was reproducible and correlated well with bolus contrast administration [47]. These findings support that ECV measurement maybe incorporated into stress perfusion protocols in both clinical and research CMR.

### The association between cardiovascular risk and cardiovascular magnetic resonance measures of fibrosis: the Multi-Ethnic Study of Atherosclerosis (MESA)

Risk scores to predict endpoints in patients without symptomatic CVD are an increasingly important tool to help identify individuals who may benefit from more intensive or earlier medical intervention. Most risk models use similar risk factors to predict cardiovascular events. These models accordingly may also be expected to show similar trends in relationship to CMR fibrosis. In this study, greater CVD risk by most risk scores was associated with greater myocardial fibrosis as identified by CMR in men [48]. T1 times showed improved relationship to CVD risk scores, compared to other CMR measures such as ECV, native and 12 min T1 time. For women, who are generally categorized as lower risk than men in CVD models, there was generally little or no relationship between CVD risk score and T1 time or ECV.

### Characterization of myocardial T1-mapping bias caused by intramyocardial fat in inversion recovery and saturation recovery techniques

Measurement of the myocardial T1 by current T1-mapping methods that use b-SSFP protocols is subject to bias when there is a mixture of myocardium and fat. The presence of fat leads to a bias in T1 measurement. Partial volume of myocardium and fat occurs at tissue boundaries and in cases of intramyocardial fat. Intramyocardial fat is frequently present in scar tissue such as chronic MI due to the process of lipomatous metaplasia, and is also present in non-ischemic cardiomyopathies. In this study, simulations were performed to illustrate the behavior of mono-exponential fitting to bi-exponential mixtures of myocardium and fat with varying fat fractions [49]. Both inversion recovery and saturation recovery imaging protocols using balanced steady state free precession were considered. In-vivo imaging with T1-mapping, water/fat separated imaging, and late enhancement imaging was performed on subjects with chronic myocardial infarction. In cases of lipomatous metaplasia, the T1 biases in b-SSFP imaging protocols were found to be additive or subtractive depending on whether the center frequency corresponds to the myocardium and fat being in-phase or out-of-phase, respectively. It is important to understand this mechanism which may otherwise lead to erroneous interpretation.

### Extracellular volume quantification in isolated hypertension - changes at the detectable limits?

Arterial hypertension results in increasing arterial stiffness and afterload, leading to remodelling of the myocardium due to cardiomyocyte hypertrophy, fibroblast stimulation and then increased collagen formation. Progressive diffuse myocardial fibrosis and left ventricular hypertrophy are recognised. In this study of well-controlled hypertensive patients from a specialist tertiary centre, of 46 hypertensive patients and 50 healthy volunteers, native myocardial T1 was similar in hypertensives and controls (955 ± 30 ms versus 965 ± 38 ms, *p* = 0.16) [50]. The difference in ECV did not reach significance (0.26 ± 0.02 versus 0.27 ± 0.03, *p* = 0.06). In the subset with LVH, the ECV was significantly higher (0.28 ± 0.03 versus 0.26 ± 0.02, *p* < 0.001). Overall, T1 mapping revealed increased diffuse myocardial fibrosis, but the increases were small and only occurred with LVH.

### Increased myocardial extracellular volume in active idiopathic systemic capillary leak syndrome

Systemic Capillary Leak Syndrome (SCLS) is a rare understudied disorder of unknown etiology, with few effective therapies, and without specific biomarkers or diagnostic tests. Using T1 mapping, it was noted in 26 patients that patients with SCLS have a significantly higher myocardial ECV than age-matched controls [51]. Elevations in ECV are most pronounced in patients with a recent SCLS flare or patients with a chronic form of SCLS. Myocardial ECV may serve as a diagnostic marker of SCLS and may aid in the evaluation of future treatments and prognosis in this rare disorder.

## T2 Mapping

Over recent years, the CMR literature has been largely dominated by work on the development and application of T1 mapping techniques. There is now renewed interest in parametric T2 based techniques that will facilitate more quantitative and sensitive assessment for myocardial oedema and inflammation than is generally achieved with conventional T2-weighted sequences. The following manuscripts suggest a number of newer techniques have the potential to provide superior quantitative T2 assessment rapidly and with high reproducibility. As with T1 mapping techniques, defining the optimal sequence parameters is paramount and reference ranges also appear to be sequence dependent.

### Cardiac T2-mapping using a fast gradient echo spin echo sequence - first in vitro and in vivo experience

This manuscript presents the work of one of the groups evaluating a novel Gradient Spin Echo (GraSE) sequence for more efficient and accurate myocardial T2 mapping [52]. They compared the more rapid newer technique with two previously introduced T2-mapping techniques (a Multi Echo Spin Echo (MESE) technique and a T2-prepared bSSFP technique) in a gel phantom and 12 healthy volunteers at 1.5 T. In the phantom study, the T2 values derived by the GraSE sequence were closer to the MESE reference than were those derived by the T2-prepared sequence; both the GraSE and T2-prepared sequences overestimated T2 when compared to MESE. This work revealed some of the potential promise of this T2-mapping sequence, while highlighting the need for systematic comparison of the different cardiac T2-mapping sequences and the requirement for sequence specific reference ranges.

### Gradient Spin Echo (GraSE) imaging for fast myocardial T2 mapping

In this manuscript, the authors also implemented and tested a novel myocardial T2 mapping sequence based on gradient-spin-echo (GraSE) to enable rapid quantitative T2 assessment with a single breath sequence at 1.5T [53]. The authors tested four sequence variants in a phantom and in 20 healthy volunteers and apply the sequence with the best performance to five patients. The authors reported impressive intra-reader and inter-reader agreement (mean differences of -0.1 ms and 0. 1ms respectively for the optimized 6-echo GraSE sequence) such that GraSE imaging may challenge previous T2 mapping techniques. The manuscript also highlights the importance of well-defined sequence settings.

### Myocardial T2 mapping reveals age- and sex-related differences in volunteers

Having performed experiments in muscle phantoms, the authors examine T2 values in 74 volunteers (including subjects with hypertension and diabetes) divided into two groups by age, using a turbo gradient spin-echo (GraSE) sequence at 1.5T [54]. As in the previous manuscript, the authors showed high reproducibility (intra- and inter-observer correlations with *R* ^2^ = 0.91 and *R* ^2^ = 0.94 as well as a coefficients of variation of 2.4 % and 2.2 %, respectively) using this technique which they found to be 6 times faster than a standard multi-echo spin-echo sequence. Quantified by freeze-drying of muscle specimens, they found myocardial T2 values to correlate with tissue water. The authors reported global T2 time to be significantly shorter towards the base of the heart (*p* < 0.01) and higher in female subjects in all short axis slices (*p* < 0.01). They found no correlation of segmental T2 values with myocardial strain, but found the older volunteers group to have significantly increased mean T2 values compared to the younger volunteers (all *p* < 0.01). This manuscript highlights age- and sex-related differences in absolute T2 values as evident in the volunteers, acknowledging that specific reference values are highly dependent on the method used such that specific reference values will be required for each T2-mapping method.

### Mapping tissue inhomogeneity in acute myocarditis: a novel analytical approach to quantitative myocardial edema imaging by T2-mapping

The authors retrospectively investigated T2-mapping data acquired using their previously described breathold Gradient-Spin-Echo sequence at 1.5 T in 31 patients with acute myocarditis and compared these to a control cohort of 30 healthy volunteers [55]. Although the authors found higher T2 values in patients than in volunteers, there was considerable overlap with mean patient values still lying within the normal range of volunteers. The authors undertook extensive statistical analysis to propose the combination of highest segmental T2 value within each patient and the mean absolute deviation of logarithm transformed pixel-standard deviation of T2 as a potential novel discriminator of patients from controls. They report that combining these enabled 83 % specificity and 81 sensitivity for detection of acute myocarditis and that this could overcome the limitation imposed by overlapping T2 values on the clinical adoption of T2-mapping, but this requires more extensive evaluation. These data further highlight the overlap between myocardial T2 values in supposedly diseased and normal myocardium, as often observed with T1-mapping.

### T2 mapping of the heart with a double-inversion radial fast spin-echo method with indirect echo compensation

The authors present a technical development in cardiac T2 mapping, detailing a black blood double-inversion recovery radial fast spin-echo (DIR-RADFSE) technique which offers superior spatial and temporal resolution to T2-SSFP methods [56]. They evaluated this sequence in phantoms, 7 healthy volunteers and two subjects with heart disease, assessing data quality and reproducibility at 1.5 T. Two different computed methods (an echo sharing (ES) algorithm and a model-based CURLIE-SEPG algorithm) for addressing the highly undersampled k-space filling approach used to limit breathhold time were compared. Both reconstruction algorithms yielded echo time images with high spatiotemporal resolution, but the accuracy and reproducibility of the CURLIE-SEPG T2 estimates were superior to those of the ES algorithm, although ES reconstruction was faster than the model-based approach. The authors suggest enhanced image quality to relate to reduced misregistration between data sets with image registration being unnecessary given that T2 mapping can be performed in a voxel-wise manner.

## Cardiomyopathy

The phenotyping of cardiomyopathy is a primary clinical indication for CMR, and has become mainstream in hypertrophic cardiomyopathy, [57–59] including the development of hypertrophy atlases [60]. Recent attention has been focussed on T1 imaging for the assessment of diffuse fibrosis in cardiomyopathy, [61, 62] with T1 standardisation, [63] technical developments, and the assessment of diffuse fibrosis in cardiomyopathy. Progress has been prominent in a wide range of unusual conditions including myocarditis, [64, 65] muscular dystrophy, [66, 67] myotonic dystrophy, [68] ARVC, [69, 70] systemic sclerosis, [71] non-compaction, [72, 73] radiation, [74] cocaine toxicity, [75] and iron loading. [76–78] This section describes further advances in this field.

### A disproportionate contribution of papillary muscles and trabeculations to total left ventricular mass makes choice of cardiovascular magnetic resonance analysis technique critical in Fabry disease

In the era of enzyme replacement therapy and the targeting of this therapy to those with identified end-organ involvement, the volumetric contribution of papillary muscles and trabeculations in Fabry disease is significantly increased relative to healthy controls in males. It is important to account for this to avert significant underestimation of LV Mass and misclassification of a proportion of subjects. While Fabry patients represent a small percentage of patients with hypertrophy, they underline the importance of accurately contouring the endocardial border for quantification of left ventricular assessment [79].

### Focal myocardial fibrosis assessed by late gadolinium enhancement cardiovascular magnetic resonance in children and adolescents with dilated cardiomyopathy

Mid-wall fibrosis using CMR is seen in adult patients presenting with non-ischemic dilated cardiomyopathy (DCM). In these studies, LGE is associated with pronounced LV remodelling and predicts adverse cardiac outcomes. The significance of these findings in a paediatric population is less clear. The purpose of this study was to determine the presence and patterns of LGE in children and adolescents with DCM [80]. The authors report that focal histologically proven myocardial fibrosis was rarely detected by LGE CMR despite marked LV dilatation and severely depressed LV function. LGE occurred in various patterns and mostly in patients with inflammatory cardiomyopathy. It remains unclear whether myocardial fibrosis in childhood DCM reflects different endogenous repair mechanisms that enable favourable reverse remodelling. Further work is required to assess the prognostic significance of LGE in children with DCM.

### Histological Validation of measurement of diffuse interstitial myocardial fibrosis by myocardial extravascular volume fraction from Modified Look-Locker imaging (MOLLI) T1 mapping at 3T

Extracellular volume fraction (ECV) measured by CMR has been proposed as a non-invasive method for assessment of diffuse myocardial fibrosis, but few studies have used 3T CMR to measure ECV. Therefore the authors aimed to validate measurement of ECV by MOLLI T1 mapping by 3T CMR against fibrosis measured by histopathology [81] The average amount of interstitial fibrosis by picrosirius red staining in biopsy samples was 6.1 ± 4.3 %. ECV computed from pre-post contrast MOLLI T1 time changes was 28.9 ± 5.5 %, and correlated (*r* = 0.78, *p* < 0.001) strongly with the magnitude of histological fibrosis. Neither the amount of LGE (*r* = 0.17, *p* = 0.36) nor native pre-contrast myocardial T1 time (*r* = −0.18, *p* = 0.32) correlated with fibrosis by histopathology. The authors concluded that ECV determined by 3T CMR T1 MOLLI images closely correlated with histologically determined diffuse interstitial fibrosis, providing a non-invasive estimation for quantification of interstitial fibrosis in patients with valve diseases.

### Characteristic cardiac phenotypes are detected by cardiovascular magnetic resonance in patients with different clinical phenotypes and genotypes of mitochondrial myopathy

Mitochondrial myopathies (MM) are a heterogeneous group of inherited conditions resulting from a primary defect in the mitochondrial respiratory chain with consecutively impaired cellular energy metabolism. This study aimed to characterize the prevalence and pattern of cardiac abnormalities and to test the additional diagnostic value of CMR in this patient population [82]. Based on assessment of 64 MM patients, cardiac involvement was a frequent finding in MM patients. Despite a high variability in clinical presentation, in patients with chronic progressive external ophthalmoplegia (CPEO)/Kearns-Sayre syndrome (KSS) patients typically showed an intramural pattern of LGE. MELAS patients were characterized by overt concentric hypertrophy and a rather unique, focally accentuated and diffusely distributed LGE.

### Comparison of cardiovascular magnetic resonance characteristics and clinical consequences in children and adolescents with isolated left ventricular non-compaction with and without late gadolinium enhancement

Left ventricular non-compaction (LVNC) is a genetically and clinically heterogeneous cardiomyopathy characterized by numerous prominent trabeculations, progressive myocardial dysfunction, malignant ventricular arrhythmias, and early mortality It accounts for about 9 % of cardiomyopathy in childhood. In this study, the authors assembled a large cohort of children with LVNC, defined by eligibility for CMR, to characterize and compare CMR features and clinical outcomes in children with LVNC with and without LGE [83]. LGE was present in up to one-fourth of children with LVNC. LGE+ children exhibited a more maladaptive LV remodeling and a higher incidence of cardiovascular death and heart transplantation.

### Abnormal septal convexity into the left ventricle occurs in subclinical hypertrophic cardiomyopathy

Approximately 60 % of cases are caused by inherited sarcomeric protein mutations. However these mutations may have incomplete expression with a variable phenotype and age-related expression. Genotyping may help identify this but is sometimes inconclusive or non-contributory. There is however, a subtle subclinical phase of HCM. In this study, septal convexity was increased in G + LVH- compared to controls with longer anterior mitral valve leaflet, higher relative wall, higher LV ejection fraction and smaller LV end-systolic volume index [84]. Other morphologic measurements (LV angles, sphericity index, and eccentricity index) were not different between G + LVH- and controls. Septal convexity is an additional previously undescribed feature of subclinical HCM.

### Cardiovascular magnetic resonance predictors of clinical outcome in patients with suspected acute myocarditis

The natural history of acute myocarditis (AM) remains highly variable and prognostic stratification is of critical importance. In a single-centre longitudinal prospective study, 203 routine consecutive patients with an initial CMR-based diagnosis of AM were followed over a mean period of 18.9 ± 8.2 months. In routine consecutive patients without severe hemodynamic compromise a number of CMR parameters including the presence and extent of myocardial edema and the extent of late gadolinium-enhanced LV myocardial lesions were not predictive of outcome [85]. The only independent CMR predictor of adverse clinical outcome was an initial alteration of LVEF.

### Cardiovascular magnetic resonance techniques and findings in children with myocarditis: a multicenter retrospective study

Viral myocarditis is an important cause of morbidity and mortality in both children and adults. This study aimed to describe the clinical presentation, CMR protocols and findings, and outcomes in a large, multicenter cohort of children with myocarditis. Despite significant practice variation in imaging protocol among centers, CMR had a high sensitivity for the diagnosis of myocarditis in pediatric patients [86]. Abnormalities were most often seen with LGE followed by T2W, EGE, and first-pass contrast perfusion. The authors note that these findings should be useful in designing future prospective studies.

### Patients with exercise-associated ventricular ectopy present evidence of myocarditis

The origin and clinical relevance of exercise-induced premature ventricular beats (PVBs) in patients without structural heart disease such as myocardial infarction, coronary artery disease, left ventricular hypertrophy, cardiomyopathies or significant valvular disease is unknown. In this study, the authors examined 162 consecutive patients presenting with palpitations and documented exercise-induced premature ventricular beats (PVBs) but no history or evidence of structural heart disease [87]. Results were compared with 70 controls matched for gender and age. ECG-triggered, T2-weighted, fast spin echo triple inversion recovery sequences and late gadolinium enhancement were obtained as well as LV function and dimensions. The majority of patients with exercise-associated premature ventricular beats present evidence of myocardial disease consistent with acute or previous myocarditis or myopericarditis.

### Cardiovascular magnetic resonance findings in patients with PRKAG2 gene mutations

PRKAG2 cardiac syndrome is an autosomal dominantly inherited metabolic heart muscle disease characterized by left ventricular hypertrophy (LVH), progressive conducting abnormalities and ventricular pre-excitation. The prevalence is approximately 0.23–1 % in patients with hypertrophic cardiomyopathy; it does however account for a greater proportion of HCM in children and adolescents. Patients may benefit from early identification due to high risk of complete atrioventricular block and sudden cardiac death caused by atrial fibrillation and rapid antegrade conduction through an accessory pathway. In this study, CMR and genetic testing were performed in two families harboring PRKAG2 mutations [88]. PRKAG2 cardiac syndrome may present with eccentric distribution of LVH, involving focal mid-infero-lateral pattern in the early disease stage, and more diffuse pattern but focusing on interventricular septum in advanced cases. In patients at earlier stages of disease, without LGE, T1 values may be reduced, while in the advanced disease stage T1 mapping may result in higher values caused by fibrosis.

### Myocardial tissue characterization in Chagas' heart disease by cardiovascular magnetic resonance

Trypanosoma cruzi infection is responsible for the Chagas’ disease (CD), a major public health issue in South America with an estimated of 13 % of Latin American population at risk of contracting this disease. With an annual incidence of 29,925 cases in 21 Latin American countries, this disease still affects approximately 5.7 million people with an average of 12,500 deaths per year. Chagas’ heart disease is the most serious complication The asymptomatic phase can last for decades, corresponding to the clinical indeterminate phase (IND), until unknown triggers initiate the progression to arrhythmias and heart failure in a subset, which represents approximately one third of the patients. Fifty-four patients were analyzed. Increase in T2-weighted (T2W) myocardial signal intensity and T1-weighted myocardial early gadolinium enhancement (MEGE) could be detected by CMR in patients throughout all phases of Chagas’ heart disease, including its subclinical presentation (IND) [89]. Moreover, findings were parallel to myocardial fibrosis (LGE) in extent and location and also correlated with the degree of Chagas’ heart disease clinical severity. These findings contribute to further the knowledge on pathophysiology of Chagas’ heart disease, and may have therapeutic and prognostic utility in the future.

### Contrast-free detection of myocardial fibrosis in hypertrophic cardiomyopathy patients with diffusion-weighted cardiovascular magnetic resonance

Previous studies have shown that diffusion-weighted cardiovascular magnetic resonance (DW-CMR) is highly sensitive to replacement fibrosis of chronic myocardial infarction. The authors evaluated this technique to detect diffuse myocardial fibrosis in hypertrophic cardiomyopathy (HCM) patients and compare its performance with established CMR techniques [90]. DW-CMR is a contrast-free non-invasive quantitative technique that was found to be sensitive to diffuse presentations of myocardial fibrosis. When compared to the established contrast-enhanced ECV-CMR, DW-CMR was able to yield comparable detection and characterization of myocardial fibrosis. DW-CMR is a promising tool but requires more robustness and shorter scan times in order for it to feasibly be an effective LGE or ECV contrast-free alternative used in a clinical setting.

### T1 at 1.5T and 3T compared with conventional T2* at 1.5T for cardiac siderosis

Myocardial black blood T2* relaxometry at 1.5T provides robust, reproducible and calibrated non-invasive assessment of cardiac iron burden. In vitro data has shown that like T2*, novel native T1 shortens with increasing tissue iron. The relative merits of T1 and T2* are largely unexplored. In this study, the authors compared the established 1.5T black blood T2* technique against native T1 values at 1.5T and 3T in iron overload patients and in normal volunteers [91]. T1 mapping at 1.5T and at 3T identified individuals with significant iron loading but there was significant scatter between results which may reflect measurement error, a T1 interaction with T2*, or a differential sensitivity to aspects of iron chemistry or other biology. Hurdles to clinical implementation of T1 include the lack of calibration against human myocardial iron concentration, no demonstrated relation to cardiac outcomes, and variation in absolute T1 values between scanners, which makes inter-centre comparisons difficult. The relative merits of T1 at 3T versus T2* at 3T require further consideration.

### Free-breathing T2* mapping using respiratory motion corrected averaging

Pixel-wise T2* maps based on breath-held segmented image acquisition are prone to ghost artefacts in instances of poor breath-holding or cardiac arrhythmia. Single shot imaging is inherently immune to ghost type artefacts. The authors proposes a free-breathing method based on respiratory motion corrected single shot imaging with averaging to improve the signal to noise ratio [92]. This demonstrated consistently good quality maps in the presence of respiratory motion and arrhythmias.

### Free-breathing myocardial T2* mapping using GRE-EPI and automatic Non-rigid motion correction

Measurement of myocardial T2* is widely used to assess patients for cardiac iron overload. The conventional breath-hold, ECG-triggered, segmented, multi-echo gradient echo (MGRE) sequence used for myocardial T2* quantification is sensitive to respiratory motion and yields artefacts in patients who are unable to breath-hold. The authors proposes a free-breathing myocardial T2* mapping approach that combines a single-shot gradient-echo echo-planar imaging (GRE-EPI) sequence for T2*-weighted image acquisition with automatic non-rigid motion correction (MOCO) of respiratory motion between single-shot images [93]. The free-breathing approach enabled accurate myocardial and liver T2* measurements and was insensitive to respiratory motion.

### Myocardial T2* mapping: influence of noise on accuracy and precision

Pixel-wise, parametric T2* mapping is emerging as a means of automatic measurement of iron content in tissues. It enables quick, intuitive interpretation and provides the potential benefit of spatial context between tissues. However, pixel-wise mapping uses much lower SNR data to estimate T2* when compared to region-based mapping thereby decreasing both its accuracy and precision. In this study, the effects that noise has on the precision and accuracy of pixel-wise T2* mapping were investigated [94]. Non-linear regression with automatic truncation was shown to be the best mapping technique for improving accuracy and precision in low T2* and low SNR measurements. A formulation for estimating pixel-wise standard deviation (SD) maps for T2* that can serve as a quality map for interpreting images and for comparison of imaging protocols was also proposed and validated.

## Perfusion

The use of CMR to assess myocardial perfusion has grown substantially, including assessment of global perfusion reserve, [95] perfusion quantification, [96] and comparison with fractional flow reserve [97]. Ongoing development continues as described in this section.

### Feasibility of high-resolution quantitative perfusion analysis in patients with heart failure

Assessment of myocardial perfusion in heart failure (HF) can be challenging due to left ventricular remodelling and wall thinning, coexistent scar and respiratory artefacts. This study assessed the feasibility of CMR stress perfusion in 58 patients with HF (LVEF ≤ 50 %) and suspected coronary artery disease [98]. All subjects underwent quantitative first-pass adenosine stress perfusion imaging using a high-resolution 3T kt perfusion sequence and voxel-wise Fermi deconvolution. Over half of the HF subjects had underlying ischaemic aetiology. Perfusion abnormalities were seen amongst patients with ischaemic HF, whilst no regional perfusion defect was observed in the non-ischaemic HF group. Good agreement was found between visual and quantitative analysis by absolute stress perfusion rate, myocardial perfusion reserve (MPR) and endocardial-epicardial MPR ratio. These results demonstrate the feasibility of high-resolution voxel-wise perfusion assessment in patients with HF.

### Quantification of myocardial perfusion with self-gated cardiovascular magnetic resonance

Current myocardial perfusion measurements are ECG-gated, but its use can be hindered when there is poor ECG signal. The authors tested a newly developed ungated perfusion sequence made from continuously acquired datasets which were retrospectively binned ("self-gated") into the systole and diastole using an iterative spatio-temporal reconstruction model [99]. The ungated acquisition was compared against the same sequence with ECG-gating as a ground truth in 7 controls. Regional myocardial blood flow estimates (MBFs) obtained using self-gated systole, self-gated diastole and ECG-gated scans were similar in these normal subjects at rest. The self-gated technique for quantification of regional myocardial perfusion could contribute to making quantitative CMR more accessible.

### Measurement of myocardial blood flow by cardiovascular magnetic resonance perfusion: comparison of distributed parameter and Fermi models with single and dual bolus

Mathematical modeling of CMR perfusion data allows quantification of myocardial blood flow. Fermi deconvolution is a popular model but overestimates myocardial blood flow in single bolus analysis due to arterial input function saturation. Dual bolus injection protocols have been suggested to eliminate saturation but are much less practical in the clinical setting. The distributed parameter model was evaluated in this study, and showed no significant difference in myocardial blood flow between single and dual bolus analysis in volunteers [100]. In patients with coronary artery disease, distributed parameter modeling was able to detect reduced myocardial blood flow at stress in all 12 stenotic vessels compared to 9 for Fermi modeling. Comparison of single bolus versus dual bolus values suggests that distributed parameter modeling is less dependent on arterial input function saturation than Fermi modeling. Distributed parameter modeling showed excellent accuracy in detecting reduced myocardial blood flow in all stenotic vessels.

### Optimization of dual-saturation single bolus acquisition for quantitative cardiac perfusion and myocardial blood flow maps

Through a series of in vitro and in vivo experiments, a dual-saturation acquisition strategy was compared to the standard dual-bolus strategy for quantification of myocardial blood flow. Adequate flow estimation by the dual-saturation strategy was achieved with myocardial tissue saturation times around 100ms (always <30ms of AIF), with the lowest echo time, and following a signal model for contrast conversion that takes into account the residual R2* effect and profile ordering. With these settings, there was a good correlation and agreement between myocardial perfusion quantitation by dual-saturation and dual-bolus techniques (R2 = 0.92). The dual-saturation acquisition strategy produces accurate estimates of absolute myocardial perfusion, and may be applied with minimal interference in standard clinical procedures [101].

### Quantitative three-dimensional myocardial perfusion cardiovascular magnetic resonance with accurate two-dimensional arterial input function assessment

Quantification of myocardial perfusion from first-pass CMR at high contrast agent (CA) dose requires separate acquisition of blood pool and myocardial tissue enhancement. In this study, a dual-sequence approach interleaving 2D imaging of the arterial input function with high-resolution 3D imaging for myocardial perfusion assessment was presented and validated for low (0.025 mmol/kg) and high (0.1 mmol/kg) CA dose [102]. Most robust myocardial blood flow estimation was achieved using the arterial input function extracted from the 2D image at high CA dose. Interleaving 2D imaging for arterial input function assessment enables robust quantitative 3D myocardial perfusion imaging at high CA dose.

### Myocardial perfusion is impaired in asymptomatic renal and liver transplant recipients: a cardiovascular magnetic resonance study

CMR was used to assess silent myocardial ischemia and epicardial CAD in renal transplant recipients [103]. Twenty renal transplant (RT) patients with no known CAD, fifteen liver transplant (LT) controls without prior CKD or known CAD, and ten hypertensive (HT) controls underwent CMR stress perfusion and coronary angiography. Myocardial perfusion reserve was significantly lower in the RT and LT groups compared to HT controls, both in the subendocardium and subepicardium. Seven (35 %) RT and five (33 %) LT had significant epicardial CAD compared to none in HT controls. One RT and one LT had LGE suggesting subendocardial infarction. RT recipients had impaired myocardial perfusion independent of LVH or diabetes mellitus. The impaired myocardial perfusion in RT was similar to LT without prior renal disease, thus unlikely related to previous CKD. It is not fully explained by the presence of significant epicardial CAD, and therefore most likely represents microvascular CAD.

### Individual component analysis of the multi-parametric cardiovascular magnetic resonance protocol in the CE-MARC trial

The CE-MARC study used a multi-parametric CMR protocol assessing 4 components: i) left ventricular function; ii) myocardial perfusion; iii) viability [late gadolinium enhancement (LGE)] and iv) coronary magnetic resonance angiography (MRA). In this pre-specified sub-study, the authors assessed the diagnostic accuracy of the individual CMR components and their combinations [104]. The full multi-parametric protocol had the highest sensitivity and was the optimal approach to rule-out significant CAD. The LGE component alone was the optimal rule-in strategy. The inclusion of coronary MRA provided no additional benefit when compared to the combination of perfusion/function/LGE.

### A review of 3D first-pass, whole-heart, myocardial perfusion cardiovascular magnetic resonance

A comprehensive review was undertaken of the methods available for 3D whole-heart first-pass perfusion (FPP) and their application to date, with particular focus on possible acceleration techniques [105]. Following a summary of the parameters typically desired of 3D FPP methods, the review explains the mechanisms of key acceleration techniques and their potential use in FPP for attaining 3D acquisitions. Although many 3D FPP methods are too early in development for the type of clinical trials required to show any clear benefit over current 2D FPP methods, the review includes the small but growing quantity of clinical research work already using 3D FPP, alongside the more technical work.

### Impact of arrhythmia on diagnostic performance of adenosine stress CMR in patients with suspected or known coronary artery disease

This study evaluated the influence of arrhythmias on the diagnostic performance of adenosine stress CMR [106]. Of the 159 patient enrolled, 72 had suspected CAD and 87 had known CAD. Diagnostic accuracy of adenosine stress CMR for detection of significant CAD was 73 % for the entire population. Diagnostic accuracy was 75 % in patients with suspected CAD, and 74 % in the group with known CAD. For different types of arrhythmia, diagnostic accuracy of CMR was 70 % in the atrial fibrillation group, and 79 % in patients with ventricular extrasystoles. The present data demonstrate good diagnostic performance of adenosine stress CMR for detection of significant coronary stenosis in patients with arrhythmia for work-up of suspected CAD or known CAD.

### Quantification of myocardial blood flow with cardiovascular magnetic resonance throughout the cardiac cycle

The aim of this study was to determine if CMR quantitative myocardial perfusion imaging accurately track physiological variations in myocardial blood flow (MBF) throughout the cardiac cycle in 30 healthy volunteers [107]. The study showed that quantitative perfusion CMR can be used to non-invasively assess cyclic variations in MBF throughout the cardiac cycle and that estimates of stress MBF followed the expected physiological trend, peaking at end-diastole and falling steadily through to end-systole. This technique may be useful in future pathophysiological studies of coronary blood flow and microvascular function.

## Stress ventriculography

### Mechanism of decreased sensitivity of dobutamine associated left ventricular wall motion analyses for appreciating inducible ischemia in older adults

Dobutamine associated left ventricular (LV) wall motion analysis exhibit reduced sensitivity for detecting inducible ischemia in individuals with increased LV wall thickness. This study was performed to better understand the mechanism of this reduced sensitivity in an elderly population [108]. All participants with inducible LV wall motion abnormalities (WMAs) had perfusion defects (PDs). However, 60 % of the participants who exhibited PDs had no inducible WMAs on dobutamine. Among these participants, myocardial oxygen demand remained lower after accounting for LV afterload and contractility, but not for either LV end-diastolic volume (LV preload) or resting concentricity. In conclusion, despite achieving target HR, a subset of elderly patients with risk factors for CAD who undergo dobutamine stress experience perfusion abnormalities without a concomitant LV WMAs. Mechanistically, this occurs in part due to a lower myocardial oxygen demand that appears related to reductions in LV preload and increases in LV concentricity.

### Downstream clinical consequences of stress cardiovascular magnetic resonance based on appropriate use criteria

The value of the most recent appropriate use criteria (AUC) was tested in 300 consecutive patients referred for CMR stress testing [109]. The results showed that 49.7 % of stress CMRs were considered “appropriate”, 36.7 % “maybe appropriate”, and 13.6 % “rarely appropriate”. Ischemia was significantly more likely in the “appropriate” and “maybe appropriate” groups than the “rarely appropriate” group. No patients undergoing catheterization in the “rarely appropriate” group went on to require revascularization, in contrast to 53.3 % of the “appropriate” and 36.4 % of the “maybe appropriate” patients. The “rarely appropriate” group never required revascularization, suggesting suboptimal resource utilization. Studies classified as “maybe appropriate” had similar rates of abnormal findings and led to similar rates of downstream catheterization and revascularization as those that were deemed “appropriate”. This suggests that consideration could be given to upgrading some of the common “maybe appropriate” indications to the appropriate category.

### Assessment of cardiovascular physiology using dobutamine stress cardiovascular magnetic resonance reveals impaired contractile reserve in patients with cirrhotic cardiomyopathy

Liver cirrhosis has been shown to affect cardiac performance, which may only be revealed under stress conditions. The authors performed a comprehensive CMR analysis of systolic function and response to dobutamine in 36 patients with cirrhosis and 8 controls [110]. Whilst volumetric and deformation parameters were similar between patients and controls at rest, patients with cirrhosis had a smaller increase in ejection fraction and cardiac output during stress. This was paralleled by an impaired improvement in circumferential, radial and longitudinal strain as assessed by feature tracking. No perfusion defects or late gadolinium enhancement were identified in any patient. In conclusion, cirrhotic cardiomyopathy is characterized by an impaired cardiac pharmacological response that can be detected by CMR stress testing.

### Prognostic utility of cardiovascular magnetic resonance upright maximal treadmill exercise testing

One hundred and fifteen (115) men and women with known or suspected coronary arteriosclerosis underwent an upright treadmill exercise CMR stress test in which left ventricular wall motion abnormalities (LVWMA) were identified before and immediately after exercise [111]. All participants completed the testing protocol, with 90 % completing image acquisition within 60 s of exercise cessation. The addition of CMR imaging identified those at risk for future events (*p* = 0.002), as opposed to the electrocardiogram stress test alone (*p* = 0.63). The results indicate that it is feasible to perform CMR wall motion assessment immediately after upright maximal treadmill exercise in patients with or suspected of coronary artery disease. The presence of inducible LVWMA during treadmill exercise stress CMR supplements ST segment monitoring and helps identify those at risk of the future combined endpoints of myocardial infarction, cardiac death, and unstable angina warranting hospitalization.

## Myocardial infarction

CMR is widely used to study patients with ischaemic heart disease and is particularly valuable to delineate myocardial infarction using late gadolinium enhancement, although more recently non-contrast techniques to detect infarction have been described [112]. The use of techniques to characterise components of myocardial injury, [113] myocardial edema, myocardium at risk, myocardial hemorrhage, [114] microvascular obstruction [115] and hibernation, [116] has also grown.

### Time elapsed after contrast injection is crucial to determine infarct transmurality and myocardial functional recovery after an acute myocardial infarction

The aim was to determine in acute and chronic MI whether the imaging time after contrast injection influences the LGE size that better predicts functional recovery [117]. Subjects were evaluated by cardiovascular magnetic resonance (CMR) in the first week (*n* = 60) and 3 months (*n* = 47) after a percutaneously revascularized STEMI. In acute MI, LGE volume decreased with longer imaging times after contrast injection; however, LGE volume remained constant over time in chronic MI. Depending on the imaging time, a change in the transmurality index was also observed in the acute phase. Infarct transmurality at 25 min post-contrast injection better predicted infarct size, functional recovery and remodelling at follow-up.

### Left ventricular global function index assessed by cardiovascular magnetic resonance for the prediction of cardiovascular events in ST-elevation myocardial infarction

This study investigated the relationship between the left ventricular global function index (LVGFI) and infarct characteristics as well as prognosis in a large multicenter STEMI population [118]. LVGFI is defined as (left ventricular stroke volume/left ventricular global volume) x 100. The median LVGFI was 31.2 %. Patients with LVGFI < median had significantly larger infarcts, less myocardial salvage, a larger extent of microvascular obstruction, higher incidence of intramyocardial hemorrhage and more pronounced LV dysfunction. MACE and mortality rates were significantly higher in the LVGFI < median group. The LVGFI had an incremental prognostic value in addition to LVEF for prediction of all-cause mortality, offering prognostic information beyond traditional cardiac risk factors.

### T1 mapping and T2 mapping at 3T for quantifying the area-at-risk in reperfused STEMI patients

Eighteen STEMI patients underwent CMR post primary percutaneous coronary intervention using native T1 and T2 mapping at 3T [119]. Matching short-axis T1 and T2 maps covering the entire left ventricle (LV) were assessed by two independent observers using manual, Otsu and 2 SD thresholds. The acquisition times were identical for the T1 and T2 maps. The Otsu thresholding technique performed best in terms of inter- and intra-observer variability for both T1 and T2 mapping CMR. There was no difference in either the mean area at risk (AAR) or myocardial salvage index between the T1 and T2 mapping techniques. On a per-slice and per-patient analysis, there was an excellent correlation between T1 mapping and T2 mapping in the quantification of the AAR with no bias. T1 mapping CMR at 3T performed as well as T2 mapping in quantifying the AAR and assessing myocardial salvage in reperfused STEMI patients, thereby providing an alternative CMR measure of the AAR.

### Unrecognized myocardial infarctions assessed by cardiovascular magnetic resonance are associated with the severity of the stenosis in the supplying coronary artery

This prospective multicenter study included 235 patients with stable angina and no history of myocardial infarction scheduled for coronary angiography [120]. The main goal was to investigate the prevalence of unrecognized myocardial infarction (UMI) by LGE CMR imaging prior to coronary angiography. UMIs were found in 25 % of patients. There was a strong association between significant stenotic lesions (≥70 % stenosis) in a coronary artery and the presence of an UMI in the myocardial segments supplied by the stenotic artery. Over half of the UMIs were located in the inferior and inferolateral myocardial segments, despite predominance for stenotic lesions in the left anterior descending artery. In summary, UMI is common in patients with stable angina and the results indicate that the majority of the UMIs are of ischemic origin due to severe coronary atherosclerosis. In contrast to what is seen in recognized myocardial infarctions, UMIs are predominately located in the inferior and inferolateral myocardial segments.

## Vascular disease

Other than its research role in vessel wall disease, CMR is mainly used clinically for the carotid arteries, [121] where it can be used to characterize plaque components, [122, 123] plaque inflammation, [124] the carotid wall, [125, 126] and disease progression [127]. MR angiography is also making progress [128–130]. CMR is widely used for assessment of the aorta in both congenital, genetic, and acquired conditions and is particularly well suited to assessment and longitudinal follow-up of aortic dimensions, [131] outcomes, [132] and more complex aspects of aortic function such as stiffness, [133] and stretch [134].

### Site-specific association between distal aortic pulse wave velocity and peripheral arterial stenosis severity: a prospective cardiovascular magnetic resonance study

Atherosclerotic vascular disease is a diffuse pathological process. Expression of the disease in one location may not represent disease severity in other vascular territories although strong correlation between disease expression and severity within the same vascular segment may be expected. The authors prospectively compared the association between distal aortic stiffness measured by CMR pulse wave velocity (PWV) with the severity of peripheral arterial occlusive disease [135]. Atherosclerotic markers sampled in remote vascular territories such as PWV in the proximal aorta and left common carotid artery wall thickness was also compared. Distal aortic wall stiffness is more directly related to peripheral arterial stenosis severity than markers from more remote vascular territories such as proximal aortic wall stiffness or carotid arterial wall thickness. Site-specific evaluation of vascular disease may be required for full vascular risk estimation.

### Evaluation of 3D multi-contrast joint intra- and extracranial vessel wall cardiovascular magnetic resonance

Multi-contrast vessel wall CMR has demonstrated its capability for atherosclerotic plaque morphology measurement and component characterization in different vasculatures. However, limited coverage and partial volume effect with conventional two-dimensional (2D) techniques might cause lesion underestimation. Therefore the authors aimed to evaluate the performance in a) blood suppression and b) vessel wall delineation of three-dimensional (3D) multi-contrast joint intra- and extracranial vessel wall imaging at 3T [136]. Three multi-contrast 3D black blood (BB) sequences with T1, T2 and heavy T1 weighting and a custom designed 36-channel neurovascular coil covering the entire intra- and extracranial vasculature were investigated. The 3D multi-contrast images acquired within 15 mins allowed the vessel wall visualization with 0.8 mm isotropic spatial resolution covering intra- and extracranial segments. Quantitative wall and lumen SNR measurements for each sequence showed effective blood suppression at all selected locations (*p* < 0.0001). Although the wall-lumen CNR varied across measured locations, each sequence provided good or adequate image quality in both intra- and extracranial segments. The authors concluded that the proposed 3D multi-contrast vessel wall technique provided isotropic resolution and time efficient solution for joint intra- and extracranial vessel wall CMR.

### Inter-study reproducibility of interleaved spiral phase velocity mapping of renal artery haemodynamics

Qualitative and quantitative assessment of renal blood flow is valuable in the evaluation of renal and renovascular diseases as well as in heart failure. In this study a high temporal resolution interleaved spiral phase velocity mapping was developed at 3T to study temporal patterns of flow and to measure renal flow resistive and pulsatility indices which are measures of downstream resistance [137]. Inter-study and interobserver reproducibility of each variable was also assessed. High temporal resolution breath-hold spiral phase velocity mapping was shown to allow reproducible assessment of renal resistive and pulsatility indices as well as renal artery blood flow. Limitations of the this study include absence of compassion of this technique with other methods currently employed, the limited spatial resolution for vessel cross-sectional areas measurements and the study cohort were relatively young volunteers only.

### Effects of age and smoking on endothelial function assessed by quantitative cardiovascular magnetic resonance in the peripheral and central vasculature

Vascular reactivity was evaluated by CMR in young and elderly non-smoker and smoker cohorts using cuff-induced hyperemia via time-resolved blood flow velocity and oxygenation (SvO2) in the femoral artery and vein, respectively. SvO2 dynamics yielded washout time (time to minimum SvO2), Arterial parameters included pulse ratio, hyperemic index and duration of hyperemia [138]. In addition, pulse-wave velocity (PWV) was assessed in the aortic arch. Age and smoking status were found to be independent for all parameters and the results suggest CMR biomarkers of endothelial function to be sensitive to age and smoking independent of each other. Ultrasound-based carotid intimal-medial thickness and brachial flow-mediated dilation were measured for comparison. The authors acknowledged number of limitations in the study design and the methods used including operator-intensive data analysis which needs to be streamlined for future large-scale studies.

### Gender specific patterns of age-related decline in aortic stiffness: a cardiovascular magnetic resonance study including normal ranges

Aortic stiffness was studied in a large cohort of healthy volunteers using CMR aortic pulse wave velocity and aortic distensibility measurements to assess gender specific patterns of age-related changes [139]. Aortic stiffness increases with advancing age in both males and females but a significant sex difference in the rate of change exists, with females showing a steeper decline in aortic elasticity. As aortic stiffness is strongly related to cardiovascular events, these observations may explain the increase in cardiovascular event rates that accompanies the menopausal age in women. Due to the cross-sectional nature of the study, the actual effect of aging within an individual on aortic stiffness would not be possible to determine. Other limitations include brachial pulse pressure assessment was used for determination of aortic distensibility and not central blood pressure and the study is exclusively in Caucasian limiting its applicability to other populations.

### Estimation of aortic pulse wave transit time in cardiovascular magnetic resonance using complex wavelet cross-spectrum analysis

CMR measured aortic pulse wave velocity is a well-established method for assessing aortic stiffness. CMR offers reliable segmental measurement of arterial length but accurate transit time (TT) determination remains a challenge. The authors developed a wavelet-based method, which enables temporal localization of signal frequencies, to estimate TT from ascending and descending aortic CMR flow curves and applied it to 71 healthy subjects (29 females) [140]. The proposed method combines the robustness of Fourier-based methods to low temporal resolution with the possibility to restrict the analysis to the reflectionless systolic upslope. The results showed that CMR measurement of aortic arch flow TT using systolic upslopes resulted in a better correlation with age and applanation tonometry at carotid and femoral arterial sites. Furthermore, methods based on harmonic decomposition were less affected by low temporal resolution.

### Arterial spin labeling perfusion cardiovascular magnetic resonance of the calf in peripheral arterial disease: cuff occlusion hyperemia vs exercise

Assessment of calf muscle perfusion requires a physiological challenge. Exercise and cuff-occlusion hyperemia are commonly used methods, but it has been unclear if one is superior to the other. The authors hypothesized that post-occlusion calf muscle perfusion (Cuff) with pulsed arterial spin labeling (PASL) CMR at 3T would yield greater perfusion and improved reproducibility compared to exercise hyperemia in studies of peripheral arterial disease (PAD) [141]. Subjects exercised until exhaustion (15 NL-Ex, 15 PAD-Ex) or had a thigh cuff inflated for 5 min (12 NL-Cuff, 11 PAD-Cuff). Controls had greater perfusion than PAD independent of stressor (NL-Ex 74 ± 21 vs. PAD-Ex 43 ± 10, *p* = 0.01; NL-Cuffmean 109 ± 39 vs. PAD-Cuffmean 34 ± 17 ml/min-100 g, *p* < 0.001). However, there was no difference between exercise and Cuffmean perfusion within groups (*p* > 0.6). The authors concluded that cuff hyperemia differentiated PAD patients from controls, as did exercise stress. Cuffmean and exercise calf perfusion values were similar. Cuff occlusion hyperemia had superior reproducibility and thus might be the preferred stressor.

### Vascular and plaque imaging with ultrasmall superparamagnetic particles of iron oxide

Ultrasmall superparamagnetic iron oxide particles (USPIO) are taken up by macrophages and can be identified in vivo by CMR. T2 and T2*-weighted imaging provide a sensitive method of assessing USPIO accumulation in tissues. In this concise and well-written review, the authors address the basic concepts, developments and applications of novel USPIOS contrast agents for CMR studies of vascular inflammation and atherosclerotic plaques imaging [142].

### Real-time aortic pulse wave velocity measurement during exercise stress testing

The validity of CMR real-time aortic PWV measurements during exercise was demonstrated in healthy subjects [143]. A velocity-sensitive real-time acquisition was adapted to provide interleaved acquisition of two locations in the descending aorta at 7.8 ms temporal resolution. An automated method was used to calculate the foot-to-foot transit time of the velocity pulse wave. Comparable PWV measurements were obtained from the standard 2D gated phase contrast velocity encoding method in phantoms. Normal volunteers showed an increase in PWV with age, and an increase in PWV with exercise. The results suggest this method would be feasible for evaluation of patients with systemic aortic pathologies.

### Non-invasive measurement using cardiovascular magnetic resonance of changes in pulmonary artery stiffness with exercise

The impact of exercise on pulmonary artery stiffness was investigated in healthy subjects using CMR measured pulmonary artery pulse wave velocity and relative area change [144]. Main pulmonary artery stiffness were shown to increase in response to acute moderate exercise in healthy subjects and that CMR exercise stress offers potential non-invasive assessment of vascular function in this setting. The study has a number of limitations including both stiffness metrics are based on measurement of the cross vascular sectional area and therefore variations in the orientation and position of the imaging plane could introduce error. The PWV method is also assumes unidirectional wave propagation along the vessel, which is only the case when reflective waves are not present. In addition, short temporal resolution is a crucial factor for accurate measurement PWV considering the short path of the main pulmonary artery.

### Detection of elevated right ventricular extracellular volume in pulmonary hypertension using Accelerated and Navigator-Gated Look-Locker Imaging for Cardiac T1 Estimation (ANGIE) cardiovascular magnetic resonance

Evaluation of diffuse right ventricular fibrosis in the setting of pulmonary hypertension (PH) is challenging but clinically important. Current CMR T1 mapping techniques have limited resolution, but accelerated and navigator-gated Look-Locker imaging for cardiac T1 estimation (ANGIE) is a novel CMR sequence with spatial resolution suitable for T1 mapping of the RV [145]. The authors use this technique for assessment of RV fibrosis and compared with normal volunteers and patients with LV heart failure and reduced ejection fraction (HFrEF) without co-existing PH, independent RV dilatation and dysfunction. Pre- and post-contrast ANGIE imaging provides high-resolution ECV determination for the RV. PH is independently associated with increased RV-ECV even after adjustment for RV dilatation and dysfunction, consistent with an independent effect of PH on myocardial fibrosis.

### Left ventricular diastolic dysfunction in pulmonary hypertension predicts functional capacity and clinical worsening: a tissue phase mapping study

Pulmonary hypertension (PH) related changes in the right ventricle can lead to abnormal left ventricular mechanics. The authors implemented novel CMR tissue phase mapping (TPM) to assess radial, longitudinal and tangential LV myocardial velocities in patients with PH [146]. The results show that TPM metrics of LV diastolic function are significantly abnormal in PH. Abnormal LV E-wave velocities were found to be the only independent predictors of functional capacity and clinical worsening in a model that includes conventional metrics of biventricular function.

## Congenital heart disease

CMR of congenital heart disease is well established, although high quality acquisition and interpretation require training. CMR is used in fetuses, [147] children, [148, 149] and adults, [150–152] often as a complement to echocardiography, to avoid imaging with ionising radiation, and reduce the need for invasive diagnostic cardiac catheterisation. CMR has a proven role in coronary anomalies, [153, 154] sudden cardiac death, [155] and pulmonary arterial hypertension [156]. Four D flow imaging, which allows the visualisation of large scale vorticity and the retrospective measurement of flow in vessels within the volume covered, [157] continues to be of interest.

### Clinical validation of free breathing respiratory triggered retrospectively cardiac gated cine balanced steady-state free precession cardiovascular magnetic resonance in sedated children

The authors prospectively studied two different cine bSSFP sequences applied in 20 sedated children with congenital heart disease mean age 8 years [158]. The standard multi signal averaged was compared with a prospective respiratory triggered bSSFP sequence. There was improved image quality with respect to edge detection although there were no consequent differences in the variability of LV and RV volumes between techniques. The finding have clinical importance for CMR in free breathing or sedated children and the improved edge definition was without a penalty in total clinical scan time.

### Foetal blood flow measured using phase contrast cardiovascular magnetic resonance - preliminary data comparing 1.5T with 3.0T

CMR phase contrast blood flow quantification reconstructed with metric optimised gating is described at 2 field strengths in a small cohort of 5 late gestation (35–38 weeks) fetuses [159]. CMR flow measures were obtained in 36 of 40 target vessels. Increased signal to noise ratio allowed better visualisation in smaller pulmonary vessels at 3.0 T which improved inter-observer agreement.

### Free breathing contrast-enhanced time-resolved magnetic resonance angiography in pediatric and adult congenital heart disease

The aim of this study was to compare a free-breathing (FB) time resolved MR angiography (TRA) technique with a conventional breath-hold MRA sequence in pediatric (age > 8 years) and adult congenital heart disease (*n* = 45) patients [160]. The FB-TRA sequence combines time efficient spiral trajectories with sensitivity encoding and partial Fourier to enable data acquisition of a 3D volume every ~1.3 seconds without temporal data sharing. FB-TRA proposed technique has sharper vessel delineation and shorter imaging time (~1.3 s) but compared with conventional MRA, the technique had inferior signal-to-noise ratio, contrast-to-noise ratio, and relative contrast. The vessel diameters and diagnostic accuracy measured from the conventional and free-breathing MRAs were also comparable. The main benefit of the proposed technique is its imaging time that simplifies bolus timing albeit with a long reconstruction time requirement and allows free-breathing acquisition of MRA images in patients unable to hold their breath.

### Use of a 1.0 Tesla open scanner for evaluation of pediatric and congenital heart disease: a retrospective cohort study

The authors present their retrospective cross-sectional institutional experience of open 1.0 T CMR compared to 1.5 T CMR for paediatric CMR or adults with congenital heart disease [161]. The patients were mainly adolescents and adults (median age 17 years). Half of patients studied had congenital heart disease. Half of the experience of open CMR was due to scheduling and 16 % due to elective choice for claustrophobia or body size. 1.0 T CMR was able to answer most clinical questions and may have a role for selected patients.

### Characterization and quantification of dynamic eccentric regurgitation of the left atrioventricular valve after atrioventricular septal defect correction with 4D Flow cardiovascular magnetic resonance and retrospective valve tracking

Building on previous work applying 4D flow to LV inflow in this condition, 32 patients after repair of atrioventricular septal defect (AVSD) were studied aiming to characterise postoperative left atrioventricular valve regurgitation (LAVV) present in 8 patients [162]. CMR 4D flow measures were correlated with echocardiography and CMR derived ventricular volumes and planimetry. 4D flow with retrospective valve tracking may be helpful to accurately and directly quantify regurgitation from the potentially multiple, eccentric and non-circular shaped LAVV regurgitation jets after repair of AVSD.

### 4D flow cardiovascular magnetic resonance consensus statement

This informative consensus paper is the work of physicists, physicians and biomedical engineers, active in the development and implementation of 4D flow CMR and describes the current advantages and disadvantages of this approach [163]. 4D flow CMR enables comprehensive access to all regions, planes and directions of cardiovascular flow and can now be achieved with typical spatial resolution of 1.5 × 1.5 × 1.5 – 3 × 3 × 3 mm3, typical temporal resolution of 30–40 ms, and acquisition times in the order of 5 to 25 min. After placement of a single acquisition volume, flow through any plane may be calculated retrospectively and with good accuracy. Research and development goals to be achieved are outlined in the statement. The clinical utility of 4D flow CMR requires further evaluation in larger prospective and multicenter trials. Meanwhile consensus example clinical indications are given.

### Atlas-based analysis of 4D flow CMR: Automated vessel segmentation and flow quantification

This study aimed to develop and evaluate a fully automatic method for segmentation and analysis of 4D flow CMR data of the great thoracic vessels [164]. Improvement and automation of post processing of 4D flow is important towards moving 4D flow into clinical practice. The proposed method was evaluated in 21 subjects (11 volunteers and 10 heart failure patients with varying degrees of valvar regurgitation) mean age 67 years studied at 3T. Evaluation of the method was performed visually, and by comparison of net flow volumes in the ascending aorta obtained automatically (using the proposed method), and semi-automatically. The proposed method allows automatic 4D vessel segmentation, assessment of flow in any number of image planes and 4D flow pattern visualisation in a relatively short amount of time and without user interaction.

### Left ventricular fluid kinetic energy time curves in heart failure from cardiovascular magnetic resonance 4D flow data

Patients with heart failure (*n* = 29, NYHA class I-IV) and controls (*n* = 12) underwent 4D flow CMR [165]. The authors used a quantitative approach to global LV performance as defined by the kinetic energy within the LV volume across the cardiac cycle and separated volume into regions defined by vortical or non-vortical flow across a range of left ventricular function. Patients with heart failure exhibit higher systolic average kinetic energy compared to controls, suggesting altered intracardiac blood flow. The different KE time curves in patients may provide a new conceptual approach for heart failure classification.

### Cardiovascular magnetic resonance catheterization derived pulmonary vascular resistance and medium-term outcomes in congenital heart disease

The authors report their extensive institutional experience with pulmonary vascular resistance (PVR) measurement using hybrid CMR and fluoroscopic guided cardiac catheterisation in a cohort of patients with congenital heart disease [166]. This method was previously validated by the same group and shown to be superior to Fick’s method as CMR flow measurement in independent of assumptions. In addition to additional diagnostic value it also allows a radiation-free or minimal radiation alternative for patients. The study cohort is 149 patients (mainly children; median age 3.6 years; range 6 days to 67 years) in whom 167 hybrid studies were performed. Mid-term clinical outcomes (4.2 years) following clinical decisions that included consideration of the results of PVR assessment are described. Patients with biventricular physiology and shunt lesions were studied to decide on whether or not the shunt communication can be closed. Patients with univentricular physiology were studied to decide on the suitability for the Fontan palliation. Hybrid PVR assessment is safe and enables reliable PVR assessment for risk stratification for CHD patients. CMR/XMR catheterisation may have particular clinical value in planning and preparing Fontan completion.

### Non-invasive determination by cardiovascular magnetic resonance of right ventricular-vascular coupling in children and adolescents with pulmonary hypertension

Seventeen patients (aged 3 months to 23 years) with available conventional right ventricular-vascular coupling ratio (VVCR) data from invasive catheterisation within 14 days were studied with CMR with the aim to retrospectively study the feasibility of non-invasive CMR measures to estimate VVCR in paediatric pulmonary hypertension (PH) patients [167]. The current interest in the clinical utility of right ventricular-pulmonary arterial coupling measurements was extended to a paediatric population and correlation with vascular reactivity, a well-known surrogate marker of disease in pulmonary hypertension was also made. CMR derived VVCR correlated with the invasive VVCR measure, the indexed pulmonary vascular resistance and vascular reactivity. The small number of patients included were heterogeneous and comprised of idiopathic PAH, congenital heart disease, interstitial lung disease and left heart disease, with both pediatric and adult patients. The use of CMR derived VVCR may have significant prognostic implication and will require further study in larger numbers, restricted to children and with less heterogeneous underlying PH causes.

### Single centre experience of the application of self navigated 3D whole heart cardiovascular magnetic resonance for the assessment of cardiac anatomy in congenital heart disease

The authors describe their institutional experience of using free-breathing self-navigated 3D-CMR in 105 congenital heart disease patients mean age 23 years at 1.5T [168]. Younger age, higher heart rate, lower ejection fraction and lack of contrast medium were independently associated with reduced image quality. Future studies are needed to compare the image quality of the self-navigated 3D SSFP to the standard respiratory navigator technique.

## Electrophysiology

### Distribution of abnormal potentials in chronic myocardial infarction using a real time magnetic resonance guided electrophysiology system

The authors conducted MR-guided electrophysiology (EP) procedures in 6 pigs, 4 weeks after an induced myocardial infarction by balloon occlusion of the left anterior descending after the second diagonal branch [169]. Infarct core, grey zone and normal myocardium were imaged with a multi contrast late enhancement CMR sequence. Abnormal local electrogram (EGM) potentials at invasive electrophysiology were correlated with CMR derived structural information relating to scar and grey zone. Based on these animal data the authors conclude that abnormal potentials and associated pro-arrhythmogenicity are most prevalent in the CMR defined grey zone. The correlation of LGE CMR grey zone and abnormal potentials was highest when EP mapping was in RV pacing rather than in sinus rhythm. These data justify future investigation of the potential clinical utility of VT ablation using CMR gray zone information.

### Quantification of atrial dynamics using cardiovascular magnetic resonance: inter-study reproducibility

Sixteen healthy volunteers underwent 3 CMRs on the same day and left atrium (LA) and (right atrium) RA reservoir, conduit and contractile booster pump functions were quantified by volumetric indices as derived from fractional volume changes and strain and strain rate deformation measured from feature tracking CMR [170]. For the LA the long axis 2- and 4-chamber views were used and for the RA the 4 chamber view. There was no morning to afternoon diurnal variation in atrial function. Inter-study reproducibility was better for volumetric indexes with equal reproducibility of these measures in the LA and RA. Reproducibility for CMR feature tracking derived strain was better than for strain rate, and was better for the LA than for the RA. LA reservoir function was the most reproducible measure of LA function whether by volumetric, strain or strain rate.

### Single breath-hold 3D measurement of left atrial volume using compressed sensing cardiovascular magnetic resonance and a non-model-based reconstruction approach

The method proposed is a new approach to high spatial and temporal resolution multi-slice cine imaging of the left atrium (LA) in a single breath hold which is combined with a non-model-based 3D reconstruction [171]. Compressed sensing with rate 11 undersampling was employed to achieve a spatial resolution of 1.5x1.5 mm2 of a 6 mm slice at 30 ms temporal resolution. A total of 5 slices was acquired in a 20 second breath-hold. The sequence was validated using LA phantoms and tested on 3 patients relative to a navigated single mid-diastole phase 3D acquisition. A high intra-and inter-observer agreement was shown for maximal and minimal LA volume measurement.

### Prognostic value of pulmonary vein size in prediction of atrial fibrillation recurrence after pulmonary vein isolation: a cardiovascular magnetic resonance study

CMR assessment of pulmonary vein anatomy with first-pass contrast enhanced magnetic resonance angiography was correlated with rate of recurrence of atrial fibrillation (AF) after pulmonary vein isolation at catheter ablation in a prospective study of 71 patients with either paroxysmal, persistent or permanent AF [172]. Larger pulmonary vein size (top 10th percentile in one or more pulmonary veins), increased left atrial size and non-paroxysmal AF were independently associated with increased recurrence of AF after pulmonary vein isolation. Patients with all 3 risk factors had a very high rate of recurrent AF.

### Left atrial structure and functional quantitation using cardiovascular magnetic resonance and multimodality tissue tracking: validation and reproducibility assessment

Twenty subjects with cardiovascular events and 10 healthy subjects with total mean age 71 were assessed with multimodality tissue tracking (MTT) and CMR derived left atrial (LA) volume and function [173]. The authors compared the accuracy of LA biplane volume and function measured by MTT and in the secondary analysis compared the inter-study reproducibility for CMR derived LA volume and function parameters. The authors conclude that LA biplane volume and function is accurate and reproducible with image analysis only half as time consuming. The intra and inter reader reproducibility for MTT was good-excellent but the interstudy reproducibility was fair-good.

## Animal studies

The use of animals to study cardiac physiology and metabolism continues to contribute a small but important section to the journal, to investigate basic principles and novel ideas, recently for example in perfusion, [174, 175] flow, [176] oxygenation, [177, 178] diffusion, [179] and fibrosis [180].

### Time course of cardiometabolic alterations in a high fat high sucrose diet mice model and improvement after GLP-1 analog treatment using multimodal cardiovascular magnetic resonance

Abdesselam et al. used single voxel 1H-MR spectroscopy to measure triglyceride content in heart and liver in a high fat high sucrose diet mouse model [181]. The spectroscopic measurements were complemented with cine and myocardial perfusion CMR. The multi-parametric CMR protocols were applied longitudinally in these diet induced obese mice to demonstrate that increase in hepatic and cardiac steatosis preceded alteration of perfusion and cardiac function. The authors further showed a beneficial effect of Exendin-4 on cardiac parameters and both hepatic and cardiac steatosis, which may represent a potential therapeutic target.

### Compressed sensing to accelerate magnetic resonance spectroscopic imaging: evaluation and application to 23Na-imaging of mouse hearts

Maguire et al. evaluated the performance of Compressed Sensing accelerated MRSI, and applied this technique to obtain accelerated sodium maps of mouse hearts in vivo [182]. More specifically, this study investigated the dependence of resolution, signal-to-noise ratio (SNR) and undersampling factor on spectral quality, and showed that signal amplitudes could accurately be determined with up to 5-fold under-sampled, CS-reconstructed MRSI. Importantly, the results from this study are equally applicable to other nuclei and tissues and by no means restricted to sodium MRSI of mouse hearts.

### Two repetition time saturation transfer (TwiST) with spill-over correction to measure creatine kinase reaction rates in human hearts

^31^P-saturation transfer (ST) allows for measuring the rate of ATP generation from phosphocreatine (PCr) via creatine kinase (CK) in the myocardium, and various approaches have previously been reported to reduce the scan time requirements for these intrinsically long experiments. Schar et al validated here a two-repetition time ST (TwiST) method, which omits the acquisition with γ-ATP saturation and short repetition time, but instead assumes an intrinsic relaxation time T_1_ for PCr [183]. The pseudo-first-order rate constant k_f_ obtained with TwiST was found to be the same as the one measured with the triple-repetition time ST (TRiST) method, but the acquisition was 9 min faster. Applied to heart failure patients, TwiST agreed with previously reported significant reductions in CK k_f_ in this patient group compared to healthy subjects, albeit at significantly reduced scan times.

### Left ventricular mechanical dysfunction in diet-induced obese mice is exacerbated during inotropic stress: a cine DENSE cardiovascular magnetic resonance study

Haggerty et al. evaluated LV mechanics under both resting and stress conditions in a murine obesity model to investigate whether or not LV mechanical dysfunction associated with obesity is exacerbated with stress and manifested at earlier stages of disease compared to baseline [184]. Cine displacement encoding with stimulated echoes (DENSE) with a temporal resolution of 7.4 ms was applied at rest (baseline) and at pharmacologically induced stress longitudinally over a period of 55 weeks. They found not only that differences in left ventricular mechanics in obese mice were exacerbated under stress conditions, but also that stress CMR revealed differences earlier than investigations at rest. The authors concluded that it may be important to evaluate cardiac function in the setting of obesity under stress conditions to fully elucidate the presence of ventricular dysfunction.

### Positive contrast high-resolution 3D-cine imaging of the cardiovascular system in small animals using a UTE sequence and iron nanoparticles at 4.7, 7 and 9.4T

Trotier et al. demonstrated in mice that 3D ultra-short echo time (UTE) multi-frame MRI can generate positive contrast independent of magnetic field strength or Ultra Small Particles of Iron Oxide (USPIO) concentration with high spatial and temporal resolution [185]. Specifically, the cine-images, which provided high SNR and CNR, did not suffer from flow or motion artifacts, and allowed for visualization of coronary arteries and the aortic valve. This approach may add to the portfolio of available techniques to provide accurate cardiac function assessment in small animal models of cardiovascular disease.

### Fast T2 gradient-spin-echo (T2-GraSE) mapping for myocardial edema quantification: first in vivo validation in a porcine model of ischemia/reperfusion

Fernández-Jiménez et al. reported on the implementation and in vivo validation of a T2 gradient-spin-echo [GraSE]) for fast and accurate myocardial T2-mapping in hearts of pigs [186]. Myocardial tissue was extracted for quantification of myocardial water content at different time points post closed chest ischemia-reperfusion surgery. Compared to a standard T2-TSE method, T2-GraSE showed excellent agreement with a mean T2 relaxation time difference of only 0.1 ms. Furthermore, both techniques showed good correlations with myocardial water content. Importantly, short acquisition and no requirement for specific software for data acquisition or post-processing allows for this approach to be easily integrated into clinical routine.

### Distal coronary embolization following acute myocardial infarction increases early infarct size and late left ventricular wall thinning in a porcine model

CMR was used to investigate the effects of no-reflow on myocardial infarction and subsequent cardiac remodelling changes following distal coronary embolization (DCE) of biologically active, blood clot material in a porcine AMI model [187]. The authors showed that DCE, which was sufficient to cause angiographic no-reflow, significantly increased early infarct size without affecting global cardiac function. However, there were no significant effects on late infarct size or global or regional cardiac function. The clinical significance of the observed increases in infarct transmurality and wall thinning in the DCE group at the late time point remained uncertain and requires further investigations.

## CMR and pacemakers

The increasing number of patients with MR-conditional devices in situ continues to be accompanied by greater CMR experience and more routine scanning of such patients, [188, 189] although stress perfusion has lagged somewhat behind due to feasibility and safety concerns, notably with regard to competitive pacing. Despite much research, accurately predicting who will respond to implantation of cardiac resynchronisation therapy (CRT) has remained problematic. CMR has developed a strong evidence base for its inclusion in the selection of patients for CRT implantation, previously to predict deleterious scar, but increasingly to predict response [190, 191].

### Prediction of response to cardiac resynchronization therapy using left ventricular pacing lead position and cardiovascular magnetic resonance derived wall motion patterns: a prospective cohort study

In this prospective study, the authors evaluate CMR predictors of response to CRT in a cohort of 33 patients with 18 (55 %) responders defined by echocardiographic criteria (a reduction in LV end-systolic volume of at least 15 %) [192]. Factors evaluated included a type II mechanical contraction pattern, the combination of a type II pattern and the LV lead position in the latest contracting site (T2CL), a true LBBB ECG pattern, and scar. On multivariate analysis, T2CL was the only independent predictor of response. Although a type 2 contraction pattern has been shown to be associated with CRT response, this original assessment for a type 2 pattern combined with lead position located in the latest activated site represents an approach with compelling incremental value.

### Feasibility and safety of adenosine cardiovascular magnetic resonance in patients with MR conditional pacemaker systems at 1.5 Tesla

The authors retrospectively analyzed their experience of performing adenosine stress CMR in 24 consecutive patients with MR conditional pacemakers who underwent CMR for the evaluation of known or suspected CAD [193]. Much attention is paid to the programming strategies used during CMR stress testing. No arrhythmia occurred as a result of any of the programming choices designed to avoid competitive pacing or bradycardia as appropriate. Although the population size is relatively small, these data support the feasibility and safety of adenosine stress MR in patients with MR conditional PPMs and provide some guidance as to programming choices for stress CMR.

## Varia

The varia section contains papers of interest that do not easily fall into a larger well described category, and includes valve disease, [194] ventricular function, [195–198] mass, [199] and shape, [200] pericardium, [201] position papers, [202] safety papers, [203] summary papers, [204] reviews, [205] and errata [206, 207].

### 2015 update on acute adverse reactions to gadolinium based contrast agents in cardiovascular MR. Large multi-national and multi-ethnical population experience with 37788 patients from the EuroCMR registry

In the latest analysis of this large cohort of patients from the EuroCMR Registry, the authors present further data strongly supporting the safe use of Gadolinium based contrast agents in CMR, reporting on the frequency and severity of adverse reactions related to more than double the number of contrast doses administered compared with the 2011 publication and with only two new severe reactions [208]. Accepting the potential for significant under-reporting, only 45 acute reactions due to contrast occurred from 37788 doses administered, a rate of 0.12 % and of these 43 of the 45 were classified as mild. The authors appropriately continue to conclude, that supported by these data, Gadolinium based contrast as a safe contrast medium for CMR should no longer be considered ‘off-label’.

### Pulmonary artery to aorta ratio for the detection of pulmonary hypertension: cardiovascular magnetic resonance and invasive hemodynamics in heart failure with preserved ejection fraction

In this interesting prospective study, the authors evaluated the power of pulmonary artery (PA) diameter, and its ratio with aortic (Ao) diameter, on top of right ventricular size, function and septomarginal trabeculation (SMT) derived by CMR, to predict elevated mean pulmonary pressure measured by right heart catheterization (RHC) and also prognosis in a relatively large cohort of patients (*n* = 111) with heart failure and preserved left ventricular ejection fraction (HFpEF) [209]. Patients were classified into one of two groups, either with or without moderate/severe pulmonary hypertension (PH), based on whether mean pulmonary artery pressure (mPAP) was 30 mmHg or more by RHC. 64 % of patients were in the moderate/severe PH group by this classification. Significant differences between groups with and without moderate/severe PH were observed with respect to PA diameter (30.9 ± 5.1 mm versus 26 ± 5.1 mm, *p* < 0.001), PA:Ao ratio (0.93 ± 0.16 versus 0.78 ± 0.14, *p* < 0.001), and SMT diameter (4.6 ± 1.5 mm versus 3.8 ± 1.2 mm; *p* = 0.008). While PA diameter measurements and PA:Ao ratio showed reasonable inter-observer agreement, measurement of SMT diameter and area as well as RV free wall thickness failed to show reproducibility. The strongest correlation with mPAP was found for PA:Ao ratio (*r* = 0.421, *p* < 0.001). By ROC analysis, the best cut-off for the detection of moderate/severe PH was found for a PA:Ao ratio of 0.83 (area under curve 0.76). Over a mean follow-up of 22.0 ± 14.9 months, event-free survival was significantly worse in patients with a PA:Ao ratio ≥0.83 (log rank, *p* = 0.004), although the event rates were mainly driven by hospitalisation rate. By multivariable Cox-regression analysis, PA:Ao ratio was the only independent predictor of event-free survival (*p* = 0.003).

### Increased pericardial fat accumulation is associated with increased intramyocardial lipid content and duration of highly active antiretroviral therapy exposure in patients infected with human immunodeficiency virus: a 3T cardiovascular magnetic resonance feasibility study

In this prospective case-control study, the authors compare magnetic resonance measures of intramyocardial and pericardial fat in 27 human immunodeficiency virus (HIV) seropositive patients on highly active antiretroviral therapy (HAART) and 22 HIV seronegative volunteers matched for age, ethnicity, body mass index and other baseline characteristics [210]. The authors report that pericardial fat volume at the level of the left main coronary artery (33.4 cm^3^ vs. 27.4 cm^3^, *p* = 0.03), but not at the level of the right ventricular free wall or the thickness over the right ventricular free wall, was greater in HIV seropositive patients. Intramyocardial lipid content (assessed by magnetic resonance spectroscopy) was also higher in HIV seropositive patients and there was correlation with pericardial fat volume (*r* = 0.58, *p* < 0.0001). In multivariable analysis limited by cohort size, pericardial fat volume at the level of the left main coronary artery remained significantly associated with HIV seropositivity. Pericardial fat volume at the level of the left main coronary artery was also correlated with time since HIV diagnosis and duration of HAART. Furthermore, pericardial fat volume at the level of the left main coronary artery was greater in those with lipo-accumulation. As acknowledged by the authors, the clinical significance of the observed differences remains to be established.

### Prognostic value of cardiovascular magnetic resonance derived right ventricular function in patients with interstitial lung disease

This prospective study evaluated the prognostic value of RVEF by CMR in 76 patients with interstitial lung disease (ILD). Pulmonary hypertension was defined by right heart catheterization [211]. The median RVEF was lower in ILD patients with PH (43.1 %) compared to those without PH (53.8 %). During a mean follow-up of 386 days, 13 severe events occurred. Multivariate Cox regression analysis showed that RVEF independently predicted future events after adjusting for age, gender and right ventricular fractional area change by echocardiography. These results suggest that CMR derived RVEF could be useful for risk stratification and clinical management of ILD patients.

### Quantitative assessment of paravalvular regurgitation following transcatheter aortic valve replacement

The authors compared CMR and transthoracic echocardiography (TTE) analysis of pre-operative and post-operative aortic regurgitation in patients with severe aortic stenosis undergoing TAVI or surgical AVR [212]. Post-procedure aortic regurgitant fraction by phase-contrast velocity was higher in the TAVI group than in the surgical AVR group. When compared to CMR based quantitative analysis, TTE underestimated the degree of paravalvular aortic regurgitation, and in 48 % of the TAVI patients, the paravalvular aortic regurgitation was at least one grade more severe on CMR. Paravalvular aortic regurgitation post TAVI when assessed by TTE may in fact be more significant and may require further evaluation by CMR.

### The diagnostic value of iron oxide nanoparticles for imaging of myocardial inflammation - quo vadis?

This is an informative and well-presented review of the basic features of superparamagnetic iron oxide-based contrast agents and the use of these agents in preclinical and preliminary clinical studies of myocardial inflammation in the setting of acute myocardial infarction and myocarditis [213]. The authors highlight the translational potential of these agents and possible research applications with regard to imaging and therapy.

### Normal values for cardiovascular magnetic resonance in adults and children

A comprehensive summary of normal values of cardiovascular parameters derived from CMR studies. An invaluable reference document to keep available on the desktop [214].

### Quantification of LV function and mass by cardiovascular magnetic resonance: multi-center variability and consensus contours

The measurement of LV mass and function by CMR is very reproducible within a centre, but less is known about inter-centre variability. In this study, 7 expert readers from 7 expert centres analyzed 15 core datasets with excellent reproducibility [215]. The multicentre consensus dataset may be used for benchmarking or training.

### Fractal frontiers in cardiovascular magnetic resonance: towards clinical implementation

Many of the structures and parameters that are detected, measured and reported in CMR have at least some properties that are fractal, meaning complex and self-similar at different scales. To date however, there has been little use of fractal geometry in CMR. This review explains the fundamental principles of fractal geometry, places the fractal dimension into a meaningful context within the realms of Euclidean and topological space, and defines its role in digital image processing [216]. It summarises the basic mathematics, highlights strengths and potential limitations of its application to biomedical imaging, shows key current examples and suggests a simple route for its successful clinical implementation by the CMR community. By simplifying some of the more abstract concepts of deterministic fractals, this review invites CMR practitioners to experiment with fractal analysis as a means of developing the next generation of intelligent quantitative cardiac imaging tools.

## Abbreviations

AIF: Arterial input function
Ao: Aorta
CA: Contrast agent
CMR: Cardiovascular magnetic resonance
CRT: Cardiac resynchronisation therapy
DCM: Dilated cardiomyopathy
DENSE: Displacement encoding with stimulated echoes
DTI: Diffusion tensor imaging
DW: Diffusion weighted
ECV: Extra-cellular volume
EF: Ejection fraction
EP: Electrophysiology
FLASH: Fast low angle shot
FT: Feature tracking
GraSE: Gradient Spin Echo
HCM: Hypertrophic cardiomyopathy
HF: Heart failure
JCMR: Journal of cardiovascular magnetic resonance
LGE: Late gadolinium enhancement
LV: Left ventricle
LVH: Left ventricular hypertrophy
LVNC: Left ventricular non-compaction
MBF: Myocardial blood flow
MDCT: Multi-detector computed tomography
MI: Myocardial infarction
MIR: Magnitude inversion recovery
MPR: Myocardial perfusion reserve
MRA: Magnetic resonance angiography
MT: Magnetization Transfer
MVO: Microvascular obstruction
PA: Pulmonary artery
PET: Positron emission tomography
PH: Pulmonary hypertension
PSIR: Phase sensitive inversion recovery
PWV: Pulse wave velocity
RAS: Renal artery stenosis
RV: Right ventricle
SASHA: Saturation recovery single-shot acquisition
SCD: Sudden cardiac death
ShMOLLI: Shortened modified look-locker imaging
SNR: Signal to noise ratio
SSFP: Steady state free precession
STEMI: ST elevation myocardial infarction
TAVI: Transcatheter aortic valve implantation
ToF: Tetralogy of Fallot
TSE: Turbo spin echo
TTE: Transthoracic echocardiography
USPIO: Ultrasmall superparamagnetic iron oxide particles
WMA: Wall motion abnormality
